# The Health of School Children

**Published:** 1920-11-20

**Authors:** 


### The Health of School Children.

### STRIKING FIGURES ABOUT DENTAL DECAY.

The latest report of the Chief Medical Officer of the Board of Education covers 200 pages, but it is not a page too long, and it should be studied closely by every educational authority in the kingdom, as it undoubtedly will be by every school medical officer. Not only does it show what is already being done in the way of medical inspection and treatment, and voluntary work through Children's Care Committees and other bodies, but it indicates the way of providing conditions which check disease at the source. Notwithstanding some improvements in the past four years, there are still seriously high figures of preventable defects due to "lack of intelligent care and forethought and to ignorance and apathy." Uncleanliness and neglected eyes at birth are stated to be the obvious causes of numberless disabilities which need never have occurred. And it is a sufficiently startling statement that fully a half of the 6,000,000 school children in England and Wales are in need of dental treatment, many urgently so.

In some areas, the Chief Medical Officer says, the opinion is expressed that the dental condition of the children as revealed by the inspections of 1919 shows an improvement on previous years. In other areas, however, the condition found could not well be worse. In Sunderland the percentage of children suffering from dental defects is appalling —no less than 94.5 per cent. The report quotes a statement by an assistant school medical officer at Sheffield, who declares that "a perfectly healthy set of teeth in a child aged six, so far as my observations have proceeded, is practically non-existent, while in three out of every four children four or more teeth show well-marked decay."

#### Some Useful Hints.

On the other hand the County Medical Officer of Shropshire draws attention to the fact that the children free from dental decay at the age of five have increased from 5 per cent. before the war to 44.4 per cent. after the war. The improvement, he suggests, may be largely due to war restrictions. The amount of sugar, sweets, and jam consumed was reduced by half, the sale of new bread was prohibited, crusts were eaten—not thrown away—and less milk was drunk. "If we could impose favourable conditions of diet," he believes, "we might in a single generation get back to the comparative freedom from dental decay of 100 or 150 years ago."

The following rules for the prevention of dental decay are suggested:—

Don't drink at meal-times, and don't eat sweet or soft starchy foods between meals.

Sweets, milk, or soft starchy food should not be taken last thing at night.

Finish a meal with cleansing food, such as fruit or raw vegetables.

A considerable amount of food at every meal should be in a form requiring vigorous mastication.

The Chief Medical Officer also directs attention to the toothbrush question, and adds that there is no reason to believe that the use of the toothbrush would suffice in itself to prevent the development of dental caries. The shortage of dentists is discussed, and the report suggests that it might be made good by modifying some of the conditions militating against the recruitment of the profession, such as the expense and length of time involved in qualifying and the competition of the unregistered practitioner.

Another important section of the report deals with defective eyesight among school children and points out that a substantial proportion of it is due to defective illumination, glare, contagion, cinemas, impossible blackboards, and near-distance work.

#### School Meals.

There are some interesting statistics with regard to the provision of school meals. The total number of meals provided in 1913-14 was 14½ millions, but rose in the first year of the war to 29½ millions, and in 1919-20 dropped to 6,300,000. The number of individual children fed in the first year of the war was 422,000, but in the year under review it was only 69,000. Less than 120 education authorities found it necessary to put the Act into force. The total expenditure was £183,000, of which £25,000 was received from the parents.

